# Physiological and Biochemical Parameters of Common Duckweed *Lemna minor* after the Exposure to Tetracycline and the Recovery from This Stress

**DOI:** 10.3390/molecules26226765

**Published:** 2021-11-09

**Authors:** Magdalena Krupka, Dariusz J. Michalczyk, Jūratė Žaltauskaitė, Gintarė Sujetovienė, Katarzyna Głowacka, Hanna Grajek, Marta Wierzbicka, Agnieszka I. Piotrowicz-Cieślak

**Affiliations:** 1 Department of Plant Physiology, Genetics and Biotechnology, Faculty of Biology and Biotechnology, University of Warmia and Mazury in Olsztyn, Oczapowskiego 1A, 10-718 Olsztyn, Poland; magkrup1209@gmail.com (M.K.); darim@uwm.edu.pl (D.J.M.); kasiag@uwm.edu.pl (K.G.); jacek_c@poczta.onet.pl (M.W.); 2 Department of Environmental Sciences, Vytautas Magnus University, Universiteto 10, 46265 Kaunas, Lithuania; jurate.zaltauskaite@vdu.lt (J.Ž.); gintare.sujetoviene@vdu.lt (G.S.); 3 Department of Physics and Biophysics, Faculty of Food Science, University of Warmia and Mazury in Olsztyn, Oczapowskiego 4, 10-719 Olsztyn, Poland; grajek@uwm.edu.pl

**Keywords:** enzyme activity, chlorophyll, membrane damage, mitochondrial activity, HSP70 protein content, free radicals

## Abstract

In this study, the ability of *Lemna minor* L. to recover to normal growth, after being degraded in a tetracycline-containing medium, was extensively investigated. The plants were exposed to tetracycline (TC) at concentrations of 1, 2.5, and 10 mM. Subsequently, their physiological status was analysed against the following criteria: rate of plant growth; free radical accumulation; antioxidant enzyme activity; chlorophyll content; HSP70 protein content; cell membrane permeability, and mitochondrial activity. The study showed that duckweed can considerably recover from the damage caused by antibiotics, within a week of cessation of stress. Of the plant properties analysed, mitochondrial activity was the most sensitive to antibiotic-induced disturbances. After transferring the plants to a tetracycline-free medium, all plant parameters improved significantly, except for the mitochondrial activity in the plants grown on the medium containing the highest dose of tetracycline. In the plants treated with this antibiotic at the concentration of 10 mM, the proportion of dead mitochondria increased and was as high as 93% after one week from the beginning of the recovery phase, even after the transfer to the tetracycline-free medium.

## 1. Introduction

Water pollution is a serious global problem that makes it necessary to constantly monitor the quality of water resources. In 2015, 663 million people used unsafe drinking water, 2.1 billion people worldwide lacked access to safe water at home, and 4.4 billion lacked safe sanitation [1]. Contaminated water is a major cause of many diseases and deaths [2]; the World Health Organization indicates that more than 3.4 million people die every year due to diseases related to contaminated water [3]. In India, 80% of health problems are due to water quality [4]. Moreover, 80% of Pakistan’s population is forced to use unsafe drinking water, due to a lack of safe and healthy water sources. Anthropogenic activities cause water-borne diseases that account for about 80% of all diseases in Pakistan, and are responsible for 33% of deaths [5]. The problem of water quality is particularly relevant in developing countries, but it affects developed countries as well. The Water Quality Report for the US published in 2017 indicated that 46% of rivers, 21% of lakes, and 18% of coastal waters surveyed were considered polluted [6]. This is very disturbing data, given that most people in the US still drink water from surface systems.

Water pollution affects entire populations of both plants and animals in aquatic biotopes. The majority of this pollution is due to human activity and includes industrial, agricultural, and municipal wastes. Antibiotics are a newly identified but particularly dangerous variety of water pollutant. Their threat stems from the potential adverse effects on non-target organisms and an increased bacterial resistance [7]. In 2020, the countries with the highest antibiotic consumption rates were China (43,024 tonnes), Brazil (7533 tonnes) and the United States (6596 tonnes) [8]. China had the highest rate of antibiotic consumption, increasing by almost 300% between 2000 and 2015. European countries, due to their smaller populations, had lower levels of antibiotic consumption, but in 2020, Spain (1804 tonnes), Italy (1057 tonnes) and Germany (773 tonnes) consumed the most antibiotics.

Antibiotics are not completely metabolised in the human and animal body; in fact, most of the ingested dose is released unchanged, polluting the environment [9]. Tetracyclines are among the antibiotics most used in animal and veterinary production [10]. The widespread use of tetracycline results from its low production costs and a wide spectrum of activity [11]. However, similarly to most antibiotics, tetracycline is poorly absorbed in the digestive tract of animals, with up to 60% of the dose being excreted in faeces, and up to 30% in urine [12]. The antibiotics excreted by the animals eventually find their way to the fields, and thus they enter the groundwater. The tetracycline content in manure is about 23 mg × kg^−1^ [13], whereas in pig slurry it is about 100 mg × kg^−1^ [14]. Tetracycline is found in surface water, groundwater and drinking water. The average concentrations of tetracycline in rivers ranges from 0.021 μg × L^−1^ (Soeste River, Germany) to 13.6 μg × L^−1^ (Wangyang River, China) [15,16]. The highest concentration of tetracycline was found in Brazilian rivers (110.1 μg × L^−1^) [17]. The mean concentration of tetracycline in lakes ranged from 0.004 to 1.05 μg × L^−1^ [18,19]. Moreover, tetracycline was also detected in groundwater (0.184 μg × L^−1^) [20] and in drinking water (0.027 μg × L^−1^) [21]. Another class of antibiotics have also been detected worldwide in seawater, lake and river water, groundwater, and even drinking water. The presence of antibiotics in drinking water, even in trace amounts, is particularly dangerous. Detailed data on the presence of antibiotics in water are presented in the Supplementary Materials (Table S1). It is likely that this presence in drinking water is due to the lack of antibiotic removal capacity in municipal drinking water infrastructure and wastewater treatment plants [22]. Antibiotics and their metabolites become a significant threat to the ecosystems, as they are fed from municipal and industrial wastewater into waterways on a regular basis. The presence of antibiotics in the environment causes toxic effects in the organisms against which they are not targeted, e.g., plants. It has also been shown that tetracycline degrades chlorophyll in plants [23], disrupts the flow of electrons in photosystems [24], and causes the overproduction of free radicals and peroxidation of membrane lipids [25]. These disturbances result in the inhibition of plant growth.

*Lemna* is an excellent indicator plant for the assessment of water quality, among others, due to its ability to absorb harmful substances present in water. It accumulates heavy metals [26], nitrogen [27], phosphorus [28], and even antibiotics [29]. In ecotoxicological studies, the duckweed is recommended [30] as a test organism due to its simple structure, small size, vegetative reproduction, and short multiplication period; the doubling time for plant numbers is as low as 48 h. The high rate of *Lemna* multiplication has resulted in a wide range of its potential biotechnological applications, including biofuel [31,32], animal feed [33], and even human food production [34].

The scientific literature on *Lemna*, however abundant, does not contain data on the recovery of these plants from exposure to antibiotic-contaminated environments. Therefore, the aim of this work was to evaluate the ability of lesser duckweed to recover after growth in a liquid medium contaminated with tetracycline. High doses of tetracycline were used in this study in order to determine the upper limit of antibiotic concentration, below which plant recovery is possible. The experiments described here provide the basis for predictions of severity, as well as the durability of the damages, caused in duckweed by antibiotics. We assessed the response of duckweed (*Lemna minor* L.) to antibiotic exposure and antibiotic removal at both physiological and biochemical levels, considering the following criteria: rate of plant growth; free radical accumulation; antioxidant enzyme activity; chlorophyll content; HSP70 protein content; cell membrane permeability, and mitochondrial activity.

## 2. Results and Discussion

The effect of tetracycline present in the medium (at concentrations of 1, 2.5, and 10 mM) on the recovery of duckweed after TC removal was investigated. The plants were grown for seven days on a tetracycline-containing medium (exposure phase) and then for seven days without tetracycline (recovery phase). The recovery capacity was evaluated by assessing the physiological and biochemical parameters, including: the rate of plant growth; frond surface area; dry and fresh weight; free radical accumulation; antioxidant enzyme activity; chlorophyll content; HSP70 protein content; cell membrane permeability and mitochondrial activity.

### 2.1. Growth Parameters

**Figure 1:**
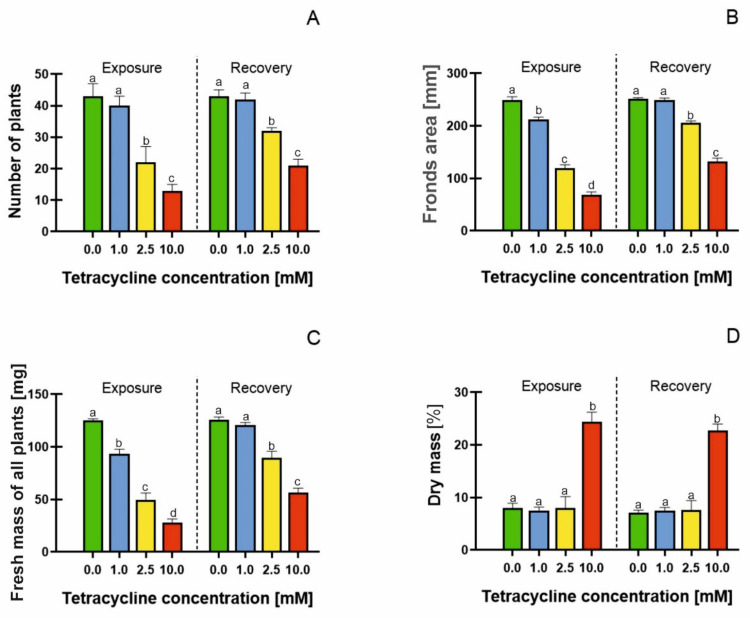
Growth parameters: number of plants (**A**), frond area (mm) (**B**), fresh mass of all plants (mg) (**C**), dry mass (%) (**D**) in *Lemna minor* subjected to tetracycline and in the recovery (resumed growth after 7 days of culture in antibiotic-free medium). Means denoted with different letters are significantly different (*p* ≤ 0.05) across the described groups: average ± standard deviation.

The OECD [30] recommends the use of growth parameters in the assessment of chemicals toxicity to *Lemna minor*. Tetracycline with a concentration range of 1 to 10 mM showed the toxicity by a reduction in the number of plants in each treatment (Figure 1A); however, the removal of tetracycline resulted in a distinct resumption of growth. Plant exposure to 2.5 mM TC caused a 48% reduction in the number of plants compared with the control, whereas the removal of TC resulted in recovery, so the ultimate reduction in plant number, at the end of the experiment, was only 8%.

Tetracycline at 10 mM concentration caused a 70% inhibition of growth at the end of the exposure phase; however, plants subjected to this treatment were also able to resume growth in an antibiotic-free medium, as culture on the medium without any TC decreased the tetracycline-induced growth inhibition by 42%. In order to discuss the recovery capacity of the lesser duckweed in more detail, the EC50 index (the concentration at which 50% inhibition of growth or other analysed parameters occurs) was determined. For the increase in *Lemna* number, this was determined as 1.944 mM tetracycline (Table 1). The EC50 parameter is usually assessed for animals [35]. The use of the EC50 parameters in this study allowed us to compare the changes in recovery capacity and recovery rate of duckweed, after the exposure to different concentrations of TC. The EC50 factor estimated for the combined TC exposure and recovery treatment was higher by 0.571 mM, compared with the value obtained before the recovery stage started. Our results clearly indicated that the EC50 parameter increased when the plant regeneration stage was added, i.e., when the tetracycline-treated plants were transferred to an antibiotic-free medium. The largest increase in EC50 (by 75%) was noted when the dry weight was analysed. The dry weight seemed, therefore, to be the plant-quality parameter which was least sensitive to tetracycline. On the other hand, the frond number was found to be the most sensitive indicator of damage caused by the temporary plant exposure to tetracycline. Adding the regeneration stage resulted in an EC50 increase of 29%, in the case of this parameter. In other words, in the case of frond numbers, the effect of the tetracycline removal was least pronounced, compared with the fronds area, fresh mass and dry mass (Table 1).

In the literature, the EC50 values for *Lemna minor* plant growth have very rarely been analysed, and the results obtained are inconsistent. The reported EC50 values for the growth of duckweed in tetracycline-containing media range from 2.38 μM [36] to 4.42 μM [29]. The difficulty in determining EC50 is probably due to the difficulty in determining the concentration at which plant death will occur. Our results indicated that tetracycline was toxic to plants even at moderate concentrations, but *Lemna* showed a good capacity to recover after the removal of the toxic agent. In plants grown in a tetracycline-containing medium, the frond surface area was reduced (Figure 1B), with particular severity in the medium containing 10 mM TC (3-fold drop compared with the control). The calculated EC50 value for frond surface area was 1.724 mM tetracycline (Table 1), which increased to 2.850 after 7 days of recovery in the tetracycline-free medium; thus, the regeneration capacity was more than 60%. Baciak et al. [29] also observed a systematic decrease in the frond area of *Lemna minor* treated with increasing concentrations of TC. Moreover, Brain et al. [37] showed that biomass is a sensitive index for the assessment of antibiotic toxicity to *Lemna* sp. The reduction in the fresh weight of the plants correlated with the accumulation of antibiotics in their tissues. Hussain et al. [38] indicated that antibiotics are accumulated in roots and leaves, and thus disrupt plant growth. Tetracycline accumulation in pea leaves was shown by Margas et al. [39]. Di Marco et al. [40] showed tetracycline accumulation in *Iberis sempervirens* L., indicating an apoplastic distribution of the antibiotic in the plant. Rydzyński et al. [41] showed tetracycline accumulation (123 μg × fresh weight ^−1^) in yellow lupin seedlings, resulting in a reduction in plant fresh weight. These results were confirmed by Rydzyński et al. [23]. The authors showed tetracycline accumulation in spinach seedlings, which correlated with a decrease in fresh weight content. Our results clearly indicate that plant fresh weight decreased significantly in samples treated with 2.5 and 10 mM tetracycline, with 60 and 76% decreases, respectively, compared with the control (Figure 1C). However, the removal of the antibiotic promoted an increase of 120% in plant fresh weight, within seven days of culture. The EC50 for fresh weight was 1.431 mM tetracycline, which increased to 2.447 mM at the end of the recovery stage of the experiment. The fresh weight was, therefore, among the investigated morphological parameters most sensitive to damage.

A significant increase in dry matter content of 300% was found in the plants treated with the highest concentration of tetracycline (Figure 1D). This change reflected the loss of water by plants, as demonstrated by the increased dry mass to frond area ratio (Figure 1B–D). The values of this ratio ranged from 0.032 in control, to 0.359 in 10 mM tetracycline treatment at the end of the exposure stage; this decreased at the recovery stage, so that it ranged from 0.028 in control, to 0.172 in 10 mM tetracycline treatment. Similar results were obtained by Rydzyński et al. [41], showing a 400% increase in dry matter of tetracycline-treated plants. The increase in dry matter content of antibiotic-treated plants was probably due to the impaired water uptake of the plant, resulting in tissue dehydration [42,43]. It may also have resulted from an increase in cell wall rigidity. Schopfer [44], in a study of maize coleoptiles, found that hydrogen peroxide inhibited the elongation of these organs and decreased the extensibility of their cell walls. He also demonstrated that the increase in cell wall rigidity resulted from the peroxidase-catalysed cross-linking of the cell wall phenolics, although the precise identification of the phenolic components was not carried out.

An analysis of the growth parameters showed that the duckweed had a high regeneration potential, when transferred to the medium without TC. Similar results were obtained by Zaltauskaite et al. [45], who treated duckweed with a sulfonylurea herbicide. The authors demonstrated that *Lemna minor* was able to regenerate after the stress factor was removed, and also indicated that the 7 days growth time in the toxicant-free medium may have been too short to achieve full-plant recovery; this is also consistent with our results. The results presented in this paper show that all growth parameters, number of plants, frond area, and fresh and dry weight, increased by about 40% after the transfer of the plants to the tetracycline-free medium, with the most visible improvements concerning the dry weight.

### 2.2. Effect of Tetracycline on Chlorophyll Content

Chlorophyll content is one of the key factors in determining plant growth. The analysis of the chlorophyll content was carried out by analysing the absorption spectra at λ = 664. According to Lamber–Beer’s law, the absorbance is described by the relation: A(λ) = ε(λ)lc, where ε(λ) is the molar extinction coefficient at wavelength λ, l is the thickness of the absorbing layer, and c is the molar concentration. The molar extinction coefficient for chlorophyll is 69 400 M^−1^ cm^−1^ in ethanol at λ = 664.7 nm, according to Seely and Jensen [46].

**Figure 2:**
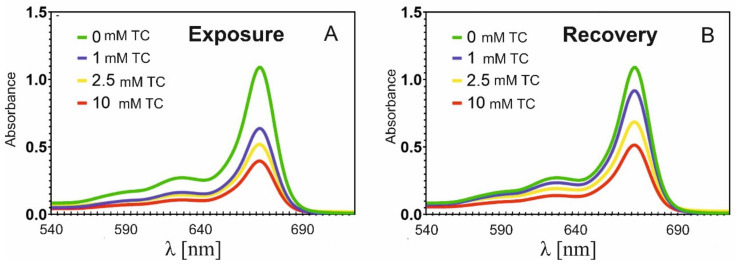
(**A**) Changes in absorption spectra of chlorophyll in *Lemna minor* subjected to tetracycline treatment and (**B**) in the recovery phase (1 week of growth in a tetracycline-free medium).

The chlorophyll content in the duckweed that was not treated with tetracycline was 1.574 × 10^−5^ M (Table 2). In the plants subjected to the lowest concentration of tetracycline (c = 1 mM) during the exposure phase, the absorbance at λ = 664 nm decreased from A = 1.09 to A = 0.64 (Figure 2A). Thus, the chlorophyll content at the end of this phase decreased to 0.918 × 10^−5^ M (Table 2). For 2.5 mM of TC, the absorbance decreased to 0.56 (chlorophyll content was 0.749 × 10^−5^), for 10 mM of TC to A = 0.37, and the chlorophyll content dropped to 0.571 × 10^−5^ M.

The reduction of the chlorophyll content in the plants subjected to tetracycline treatment has been observed repeatedly [23,41,47]. Margas et al. [47] have shown that in peas exposed to 250 mg × L^−1^ tetracycline, a 37% decrease in chlorophyll content occurred. Moreover, a decrease in the absorbance of chlorophyll was also observed by Rydzyński et al. [41], who analysed the leaves of yellow lupin growing on soil containing tetracycline. These authors showed a decrease in chlorophyll concentration in every sample tested. The reduced chlorophyll concentration was particularly evident in the young leaves. Our current results indicate that the tetracycline treatment also reduced the concentration of chlorophyll in *Lemna*, and the intensity of this effect was related to the antibiotic concentration (Table 1). However, transferring the plants to a medium without tetracycline clearly resulted in the recovery of chlorophyll absorbance (Figure 2B). The increase in chlorophyll absorbance resulted from the increase in its concentration; for plants treated with 1 mM TC, there was an increase in absorbance to A = 0.937 (up 43%), and for 2.5 mM TC to A = 0.711 (31%), whereas for 10 mM TC it only slightly increased to A = 0.552 (29%).

This demonstrates that the chlorophyll concentration in the plants growing after the tetracycline removal did not reach the same values as in the control. Therefore, it can be stated that the degradation of chlorophyll in the plants treated with pharmaceuticals was at least partially reversible, but seven days was too short a time for full recovery to occur.

As indicated by Rydzyński et al. [23], the decrease in chlorophyll content in plants treated with antibiotics may result from the direct degradation of chlorophyll, and from the formation of its degradation products. The authors conducted a study on the direct effect of tetracycline on chlorophyll, and showed shifts in the chlorophyll absorption bands as well as the appearance of new absorption bands, due to the formation of chlorophyll degradation products, including pheophytin. The model proposed by the authors for the degradation of chlorophyll by tetracycline involved the removal of a magnesium ion from the porphyrin ring. The energy transport in chlorophyll antennae was then disrupted and thus photosynthetic efficiency was reduced [23].

### 2.3. Free Radical Accumulation and Antioxidant Enzyme Activities

**Figure 3:**
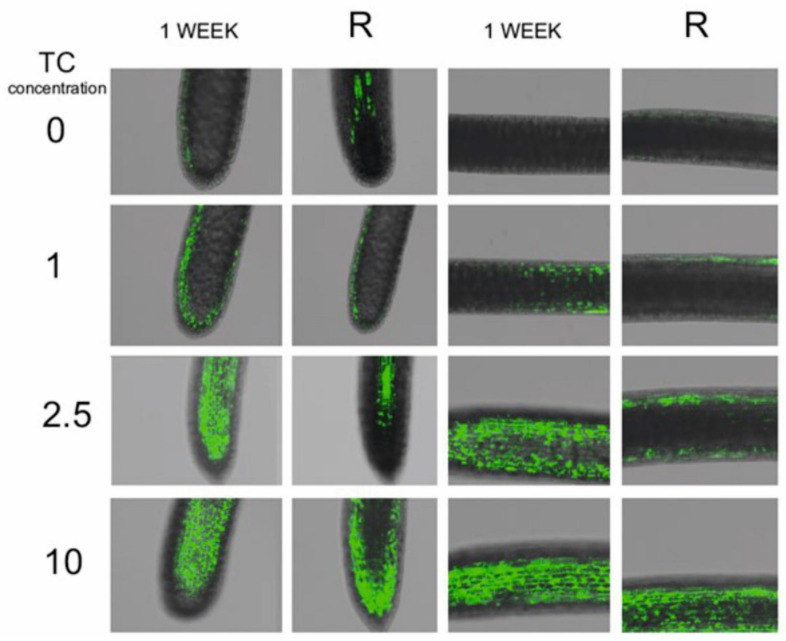
Free radicals (green colour) in roots (500×) of *Lemna minor* subjected to tetracycline (1 week) and in the recovery phase (R) (resumed growth after 7 days of culture in antibiotic-free medium).

The H_2_DCFDA was used as a marker for the total ROS production. H_2_DCFDA is taken up by the cells, then deacetylated by intracellular esterases to DCFH. DCFH can be oxidized to fluorescent DCF by hydrogen peroxide, lipid peroxides and reactive nitrogen species (RNS), and also by peroxidases even in the absence of hydrogen peroxide [48,49]. Therefore, this assay is best applied as a qualitative marker of cellular oxidant stress [50], but should be carried out in parallel with peroxidase activity [51]. Oxidative stress, leading to the formation of reactive oxygen species (ROS), lies at the origin of nearly all plant stresses. Although ROS are also formed under physiologically normal conditions, mainly as byproducts of photosynthesis and respiration, environmental stresses can cause ROS overproduction [52]. The prevalence of free radical accumulation over free radical scavenging mechanisms is the core of oxidative stress [53]. In tetracycline-treated plants, ROS accumulation was observed (Figure 3). The fluorescence intensity, associated with the presence of ROS, increased with increasing tetracycline concentration; thus, the highest ROS accumulation was shown in plants treated with the highest tetracycline concentration.

Similar results were obtained by Margas et al. [47], showing free radical accumulation in the roots of peas (*Pisum sativum* L.) treated with tetracycline, as well as Orzoł et al. [54], in the root of yellow lupin treated with levofloxacin.

To date, no studies have been conducted on the free radical accumulation in plants recovering from tetracycline treatment. After transferring the tetracycline-treated duckweed to a medium without tetracycline, a reduced accumulation of ROS was observed (Figure 3) in each test sample. However, the concentration of ROS was still higher than in the control sample. In addition, it was observed that ROS accumulated mainly in the apical part of the root, whereas during the tetracycline-exposure phase, ROS accumulated in both the apical and the middle part of the root. It is known that ROS can modify the structure of cell walls, and even determine the size of the meristematic zone of roots by regulating the cell cycle [55,56]. Differences in the spatial distribution of ROS in root tips during the recovery from the TC treatment (10 mM) may suggest the mechanism of root growth inhibition by tetracycline and the recovery from this stress [56]. Our results indicated that free radical scavenging occurred in the plants transferred to the medium without TC, suggesting the activation of free radical scavenging mechanisms. One of the mechanisms for the removal of free radicals from cells was the activation of antioxidant enzymes. Catalase (CAT) is an enzyme that directly converts hydrogen peroxide to water and oxygen [57].

**Figure 4:**
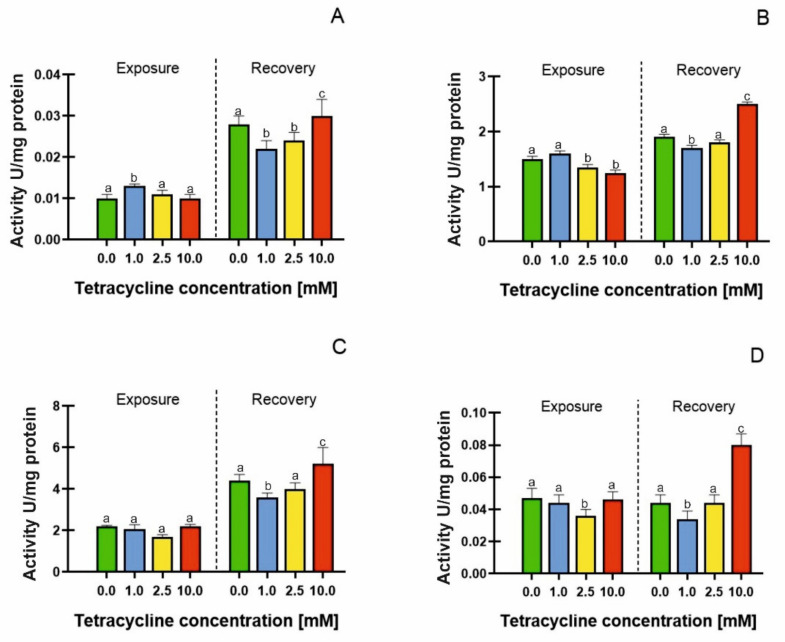
Catalase activity (**A**), ascorbate peroxidase (**B**), pyrogallol peroxidase (**C**), glutathione reductase (**D**) in *Lemna minor* subjected to tetracycline (exposure) and in the recovery phase (R) (resumed growth after 7 days of culture in an antibiotic-free medium). Means denoted with different letters are significantly different (*p* ≤ 0.05) across the described groups: average ± standard deviation.

During the exposure phase, increased catalase activity was only observed after treatment with 1 mM tetracycline in the medium: a 30% increase in comparison with the control (Figure 4A). However, a significant increase in catalase activity was observed during the recovery phase (Figure 4A); the enzyme activity increased by 120, 140, and 200%, for tetracycline 1, 2.5 and 10 mM treatments, respectively, compared with plants analysed directly after the exposure phase. The increase in catalase activity was also expressed by the EC50 parameter that elevated from 2.711 mM (at the end of the exposure phase) to 6.506 mM in the recovery phase (Table 3). Drobniewska et al. [58] carried out a similar study, investigating the recovery of *Lemna minor* L. treated with sulfadimethoxine; however, the authors did not show an increase in CAT activity during the recovery phase.

Ascorbate peroxidase (APX) is an enzyme that catalyses the decomposition of hydrogen peroxide, using ascorbate as an electron donor. This enzyme is particularly active in chloroplasts [41]. Ascorbate peroxidase (APX) activity was expressed as 1 U/mg protein. During the exposure phase, the enzyme activity decreased in the plants treated with tetracycline at concentrations of 2.5 mM (15% decrease in activity) and 10 mM (22% decrease in activity). APX was also the most sensitive antioxidant enzyme (EC50 value = 2.173 mM). Similar results were obtained by Gomes et al. [24]. These authors showed that in *Lemna minor* L. treated with ciprofloxacin, there was a decrease in APX activity with a concomitant accumulation of ROS, suggesting a clear dominance of ROS accumulation over ROS removal mechanisms.

During the recovery phase, we observed a general increase in enzyme activity (and an increase in the EC50 parameter to 5.231 mM), especially in the plants treated with the 10 mM antibiotic. In these samples, the APX activity increased by 66%, compared with the control plants (Figure 4B). Similar results were also obtained for peroxidase (PDX) (Figure 4C); however, no significant differences in peroxidase activity were observed during the exposure phase (the EC50 value for this parameter was 9.253 mM). In the recovery phase, however, a large increase in the enzyme activity was already visible in the samples treated with the lowest concentration of tetracycline (an increase in PDX activity of 66% compared with the control). In plants subjected to tetracycline at a concentration of 2.5 mM, peroxidase activity increased by 81%, whereas in plants treated with the highest tetracycline concentration, it increased by 136% compared with the control. The increase in PDX activity was correlated with a decrease in EC50 values to 5.098 mM. POX activity was higher than that of APX, though the tetracycline-induced changes in the activities of these enzymes were more notable in the case of APX. However, it could be clearly seen that the pattern of the changes was the same, and some similarities could be seen with the CAT response as well. So, our data show that TC affects hydrogen peroxide metabolism in the plant. However, no clear dependence on the TC concentration could be detected, i.e., there was no significant statistical relationship between concentration and response. Moreover, the moderate decrease in particular enzyme activity in the treatments of 2.5–10 mM TC concentrations did not necessarily indicate adverse ROS effects, because several enzymes were involved in hydrogen peroxide scavenging.

Glutathione reductase (GR) is the factor responsible for maintaining adequate levels of reduced glutathione (GSH) in the cell. Reduced GSH is responsible for the cellular control of reactive oxygen species [59]. We recorded an increase in GR activity only in the plants undergoing recovery from the 10 mM tetracycline treatment. The highest concentration of tetracycline resulted in an almost two-fold increase in GR activity in the recovery phase (Figure 4D). GR activity was mainly detected in the photosynthesising tissues. In our results, we showed a positive correlation between the GR activity in the recovery phase and the increased chlorophyll concentration. The exposure to moderate TC concentrations (1–2.5 mM) led to a gradual reduction in GR activity, though the treatment of 10 mM GR was similar to that of the control. After the recovery period, GR activity remained lower than the control only in the treatment of 1 mM TC. The GR level in the *L. minor* pre-exposed to 2.5–10 mM showed a complete recovery in this ASC/GSH cycle enzyme, indicating an efficient antioxidant defense system. A moderate recovery was also recorded in the other ASC/GSH cycle enzymes.

The increased activity of antioxidant enzymes is a good biomarker of a plant’s stress response. On the other hand, the prevalence of free radical accumulation over free radical scavenging mechanisms may lead to permanent structural damage to the plant cell. Liu et al. [25] reported that in ginger cells treated with 500 mg × L^−1^ tetracycline, an increase in malondialdehyde (MDA) concentration occured, despite the concurrent increase in CAT and PDX activity. The authors indicated that the increased enzyme activity was apparently not sufficient for free radical scavenging. Consequently, membrane lipid peroxidation occurred, leading to the accumulation of MDA as the peroxidation product. However, knowing that DCFH can be oxidized to fluorescent DCF by peroxidases even in the absence of hydrogen peroxide, it is possible that the increased amount of ROS in the roots in the recovery phase was caused by the peroxidases. 

### 2.4. Malondialdehyde (MDA) Content, Cell Membrane Damage, Mitochondrial Activity and HSP70 Protein Content

**Figure 5:**
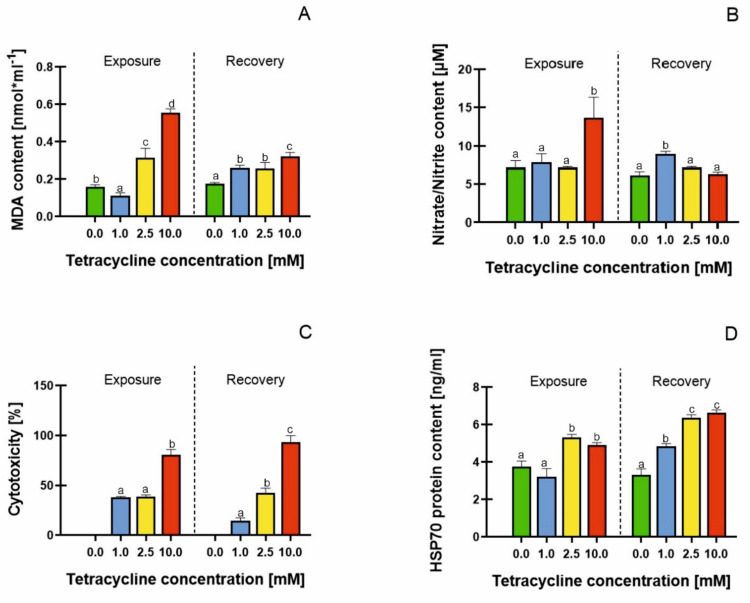
Malondialdehyde (MDA) content (**A**), cell membrane damage (**B**), mitochondrial cytotoxicity (**C**), and HSP70 protein content (**D**) in *Lemna minor* subjected to tetracycline (exposure) and in the recovery phase (resumed growth after 7 days of culture in an antibiotic free medium). Means denoted with different letters are significantly different (*p* ≤ 0.05) across the described groups: average ± standard deviation.

The MDA content measured at the end of the exposure phase increased significantly as the tetracycline concentration increased to 2.5 and 10 mM, by 196 and 346%, respectively, compared with the control (Figure 5A). Liu et al. [25] also showed lipid peroxidation in ginseng cells treated with 500 mg× L^−1^ tetracycline, reporting a 2.5-fold increase in MDA content, compared with the control. On the other hand, Nunes et al. [60] observed no significant differences in MDA content in *Lemna minor* L. and *Lemna gibba* L. treated with fluoroquinolone antibiotics. The authors suggested that both plants were able to cope with oxidative stress by eliminating ROS, thus protecting cell membranes from lipid peroxidation, probably through catalase activity. Such an effect was also demonstrated in this paper, in plants undergoing the recovery phase. We detected a decrease in MDA content of 45%, 31%, and 19%, compared with the exposure phase (Figure 5A), with an increase in antioxidant enzyme activity and a decrease in free radical accumulation.

In addition to the peroxidative damage to the cell membranes, we investigated the effect of antibiotics on cell membrane integrity by determining nitrate and nitrite. In plants, nitrate and nitrite are involved in nitric oxide synthesis. Nitric oxide (NO) is a signalling molecule responsible, among other things, for vegetative development or ion homeostasis. However, under stress conditions, NO accumulation induces toxic effects in plants [61]. During the exposure phase, a significant increase in nitrate and nitrite content occurred in plants treated with TC at the highest concentration, showing a 89% increase compared with the control (Figure 5B). Valderrama et al. [62] showed the increased production of NO and the accumulation of its derivatives in olive leaves under salt stress. The accumulation of reactive nitrogen species was demonstrated by Kummerova et al. [63] in *Lemna minor* L. treated with diclofenac. The overproduction and accumulation of NO outside cell membranes is the first symptom for the loss of membrane integrity in cells undergoing programmed cell death [64]. Therefore, it can be assumed that in plants treated with tetracycline at a concentration of 10 mM, there was a loss of cell membrane integrity and consequently the onset of cell apoptosis; however, transferring plants to a tetracycline-free medium reduced this effect by 46%.

The assessment of mitochondrial viability can be an important parameter in the evaluation of biochemical changes induced by antibiotics in plants. To assess the status of mitochondria, we used the WST-1 assay based on the cleavage of tetrazolium salt to formazan, through the activity of dehydrogenases present in mitochondrial membranes. In the control samples, we did not observe mitochondrial damage. Increasing the concentrations of tetracycline caused 37, 38 and 80% mitochondrial toxicity (Figure 5C). Kummerova et al. [63] showed a reduced mitochondrial activity in *Lemna minor* L. treated with diclofenac. Our study similarly indicated that mitochondria may be the site of antibiotic direct action in plants. The effect of antibiotics on mitochondria was also described by Geisler et al. [65], who treated potatoes with antimycin A, and showed a reduction in NADH dehydrogenase activity in leaves. Gomes et al. [66] showed that in *Lemna minor* L. treated with ciprofloxacin there was a disruption of the mitochondrial electron transport chain. These results suggested that the antibiotic-induced impairment of mitochondria may result from both structural damages in mitochondrial membranes and the disruption of electron transport. Moreover, the oxidative damage to the plant’s mitochondria may result from the accumulation of free radicals correlates with the insufficient activity of antioxidant enzymes [67]. When the duckweeds were transferred to a tetracycline-free medium, a 23% reduction in the frequency of dead mitochondria was observed. The highest concentration of tetracycline caused a 93% mitochondrial cytotoxicity, measured during the recovery phase (Figure 5C). This was the only parameter that continued to increase after the removal of TC from the medium.

HSP 70 proteins are also essential components of the plant stress response. Their main role is to protect cells from oxidative stress by stabilizing membrane proteins and removing abnormal and damaged proteins [68]. Although they accumulate in the highest amount during heat stress, they are a biomarker of the stress responses induced by other factors as well, including anthropogenic factors. HSP proteins have been identified in various cellular structures, e.g., in chloroplasts, cell nuclei, the cytoplasm, cell membranes and mitochondrial membranes [68]. HSP proteins are involved in repair processes and are a good biochemical marker of the plant’s stress response. A significant increase in HSP 70 protein content was observed when tetracycline was added to the medium at concentrations of 2.5 and 10 mM. The HSP 70 protein content in these samples was 51.86 and 48.71 ng × mL^−1^, respectively (131 and 123% increase, compared with the control). Gorovits et al. [69] also showed an increase in HSP 70 content in tomato leaves treated with pharmaceuticals, in the concentration range of 10–100 μg × L^−1^. Even the lowest concentration of pharmaceuticals (10 μg × L^−1^) induced HSP 70 proteins in leaves. In contrast, no increase in HSP 70 proteins was observed in the roots. Margas et al. [39] and Ziółkowska and Piotrowicz-Cieślak [70], on the other hand, observed a reduction in HSP 70 proteins in pea roots under the influence of pharmaceuticals. Das et al. [71] indicated that in soybeans under heat stress, increased levels of HSP 70 proteins activated a cascade of proteins responsible for protecting the photosynthetic apparatus and chloroplasts.

We observed an increase in HSP 70 protein content by 26, 25 and 34%, in the plants recovering from TC 1, 2.5 and 10 mM treatments, respectively (Figure 5D). The increase in HSP 70 content in the recovery phase correlated with a decrease in free radical accumulation, and with an increase in the chlorophyll concentration in plants. Considering the above aspects, it can be assumed that HSP 70 proteins play an important role in the repair processes in antibiotic-treated plants.

## 3. Materials and Methods

### 3.1. Plant Material and Culture Conditions

Axenic cultures of duckweed (*Lemna minor* L.) maintained in the Department of Plant Physiology, Genetics and Biotechnology of the University of Warmia and Mazury in Olsztyn, were used. Ten specimens of plants were cultured for 7 days in glass jars with the capacity of 200 mL, in 100 mL of liquid 50% Murashige and Skoog (MS) medium at 25 °C/17 °C, day/night temperatures, 16/8 h photoperiod, with daylight intensity 3.4 klx (fluorescent lamp Osram L36W/77 Fluora). Additionally, the medium contained tetracycline added at concentrations of 0, 1, 2.5 and 10 mM. After 7 days of culture in the tetracycline treatment, the plants were transferred to a fresh medium with the same content as above, only without the tetracycline (recovery phase). The plant responses were analysed after 7 days of tetracycline treatment and then again after 7 days of the recovery phase.

### 3.2. Analyses of Growth, Frond Area, Dry and Fresh Weight

The number of plants, total frond area and dry and fresh weight were determined according to the OECD protocol for *Lemna* sp. [30].

### 3.3. Isolation and Absorption Measurements of Chlorophyll

The plants (0.3 g) were homogenized in a mortar with 5 mL of 96% ethanol. After centrifugation at 1700× *g* for 15 min, the pellets were washed with the solvent five times, and the ethanol solutions from all extraction stages were combined. The supernatants were diluted five times; then, they were subjected to analyses of absorption spectra using a Carry 300 UV-Visible Spectrophotometer (Varian, Inc., Victoria, Australia) according to Rydzyński et al. [72].

### 3.4. Presence of Free Radicals (ROS)

Control seedlings and seedlings grown on tetracycline containing media were analysed to detect the presence of reactive oxygen species (ROS). To detect ROS, 2,7-dichlorodihydrofluorescein diacetate (H_2_DCFDA; Sigma-Aldrich, Poland) was used. Plants were placed in H_2_DCFDA solution in 0.1 M phosphate-buffered saline (PBS, Merck, Poland) in the dark. After 30 min, the buffer was replaced with fresh PBS. After staining, the samples were analysed by confocal laser-scanning microscopy (Leica TCS SP5) and the Leica Application Suite 2.0.2 build 2038. The following excitation and emission wavelengths were used in the experiment: 488 nm excitation and 515–565 nm emission.

### 3.5. Enzyme Activity

The plant extracts were prepared on ice. The plants were then ground in liquid nitrogen, using a porcelain mortar and pestle. For antioxidative enzymes, plants were homogenized in 0.05 M K-phosphate buffer (pH 7.0), containing 2% (*w*/*v*) PVPP, 0.4 mM EDTA, 0.2 mM PMSF by Retsch Mixer Mill MM400 (Germany). The samples were centrifuged for 20 min at 12,000× *g* at 4 °C. The supernatant was then carefully collected, and the pellet discarded.

The catalase activity was determined spectrophotometrically (SPECTROstar Nano), in a reaction mixture containing 50 mM phosphate buffer, pH 7 and 15 mM H_2_O_2_. The absorbance was measured for 10 min at room temperature, at 240 nm according to Aebi [73]. One unit corresponded to a reduction of 1 μmol H_2_O_2_ in 1 min.

The ascorbate peroxidase activity was determined spectrophotometrically in a 1 mL reaction mixture containing 50 mM potassium phosphate buffer (pH 7.0), 0.35 μM ascorbate and 10 μM H_2_O_2._ APX activity was determined by following the decrease in absorbance at 290 nm for 10 min at room temperature, according to Murshed et al. [74]. One unit corresponded to a reduction of 1 μmol H_2_O_2_ in 1 min.

Pyrogallol peroxidase activity was determined spectrophotometrically (λ = 420 nm) in a reaction mixture containing 100 μL of 1% pyrogallol (2,3-Dihydroxyphenol, Merck, Poland), 2 mL of 0.1 M 50 mM phosphate buffer, pH 6, 50 μL of supernatant and 20 μL of 0.06% H_2_O_2_. The rate of increase in absorbance was measured at room temperature at 420 nm. One unit corresponded to 1.0 mg of purpurogallin from pyrogallol in 20 s at pH 6.0 at room temperature according to Chance and Maehly [75] and Radić et al. [76].

Glutathione reductase activity was determined with a spectrophotometer (CECIL, CE2021 2000 SERIES, Cambridge, United Kingdom) in a reaction mixture containing 100 mM potassium phosphate buffer (pH 7.8), 2 mM EDTA, 0.2 mM NADPH (β-Nicotinamide adenine dinucleotide phosphate, Sigma-Aldrich, Poland) and 0.5 mM GSSG (L-Glutathione oxidized, Merck, Poland). The rate of decrease in absorbance was measured at room temperature at 340 nm according to Murshed et al. [77]. One unit corresponds to the oxidation of 1μM NADPH in 1 min.

### 3.6. Lipid Peroxidation—TBARS Assay

In order to assess lipid damage, the method of Hodges et al. [78] with modifications was used. An amount of 0.4 g of tissue was homogenized in a cold porcelain mortar and pestle (on ice) in 4 mL 0.1% trichloroacetic acid (TCA, Sigma-Aldrich, Poland). The extracts were centrifuged at 5000× *g* for 10 min. Then 1 mL of 50% ethanol solution was added, the extracts were incubated for half an hour, and centrifuged at 5000× *g* for 10 min. The procedure was repeated twice. An amount of 1 mL of supernatant was taken and a mixture of 20% trichloroacetic acid and 0.5% thiobarbituric acid (TBA, Sigma-Aldrich, Poznań, Poland) was added. The extracts were heated in a water bath at 95 °C for 30 min. The extracts were ice-cooled and centrifuged at 5000× *g* for 10 min. Absorbance was measured at 525 nm and 600 nm as a reference using a TCA/TBA mixture. The assay was performed on plants collected after the antibiotic exposure and the recovery phase. The results were presented as the content of malondialdehyde (MDA, [nmol × mL^−1^]). The MDA content was calculated according to the method of Heath and Packer [79], using the molar extinction coefficient MDA 155 mM^−1^ × cm^−1^.

### 3.7. Assessment of Damages to Cell Membranes

Cell membranes were isolated from *Lemna minor* fronds according to Jett et al. [80]. An amount of 0.4 g plant material was homogenized in a 10 mM Tris/HCl, pH 7.5 buffer, containing 2 mM EDTA. The homogenate was centrifuged at 5000× *g* for 10 min. Tris/HCl buffer, pH 7.4, containing 1 mM MgCl_2_ was added, and after 24 h the extracts were centrifuged at 100× *g* for 5 min. An amount of 5 mL aliquots from the upper and bottom phases were collected and centrifuged at 10,000× *g* for 10 min. A two-phase solution was then prepared by mixing 9.68 g of dextran (Sigma-Aldrich, Poznań, Poland) suspended in 100 mL Tris/HCl buffer, containing EDTA, pH 7.5, and 7 g of polyethylene glycol (Sigma-Aldrich, Poznań, Poland), suspended in 100 mL of the same buffer. The samples were suspended in the two-phase solution and allowed to sit at 4 °C for 24 h. After 24 h, 40 μL samples were aliquots to a 96-well multiplate. Next 20 μL assay buffer was added to each well. Ten μL of a feshly prepared 1 mM NADPH solution was added, and the plate was kept at room temperature for 60 min. After the incubation 10 μL of the lactate dehydrogenase (LDH) was added and 10 μL of a cofactor solution. Reagent 1, and the next 50 μL Griess Reagent 2 were added. The absorbances of the samples were measured at 540 nm. The standard curve was used to determine the nitrate concentration [μM].

### 3.8. Assessment of Mitochondrial Damages—WST-1 Test

Mitochondria were isolated essentially according to Heckman et al. [81]. Homogenization was carried out in a pH 7.6 buffer with the following composition: 350 mM mannitol, 30 mM Mops (3- (N-Morpholino) propanesulfonic acid sodium salt (Sigma-Aldrich, Poznań, Poland), 1 mM EDTA (Ethylenedinitrilotetraacetic acid; Merck, Warsaw, Poland) with the addition of 1.8 g of insoluble PVPP (Polyvinylpolypyrrolidone; Merck, Warsaw, Poland) and 0.34 g of L-cysteine in 100 mL. An amount of 0.4 g of tissue was ground in 5 mL of ice-cold buffer in a mortar. The homogenate was filtered and then centrifuged at 4732× *g* for 2 min at 4 °C. The supernatant was removed and centrifuged at 18.207× *g* for 5 min at the same temperature. The supernatant was discarded, and the pellet was washed with 1 mL of a pH 7.2 buffer containing 300 mM mannitol, 20 mM Mops and 1 mM EDTA. After a 2 min centrifugation at 4732× *g* the supernatant was transferred to new tubes, and a 0.6 M sucrose solution was added. The samples were centrifuged at 9583× *g* for 20 min. The supernatant was discarded, and the pellet was dissolved in pH 7.2 buffer, containing 250 mM sucrose and 30 mM Mops.

An assessment of mitochondrial damages was performed using the cellular cytotoxicity assay with the WST-1 reagent (Cayman Chemical). An amount of 100 μL of sample and 10 μL of WST-1 reagent solution were placed in a 96-well plate. The plate was incubated at 37 °C (the optimum temperature for conversion of tetrazolium salt to formazan) for 2 h. The absorbance of the samples was measured at 440 and 620 nm. The 620 nm absorbance was used to correct the readings for the natural hew of the extracts. The absorbance of the unheated sample was used as a blank. Mitochondrial cytotoxicity was calculated according to the formula:

(1) $$cytotoxicity=\frac{Abscontrol-Abssample}{Abscontrol}\times 100\%$$

where: Abs control = the reference wavelength at 620 nm recommended by manufacturers of some cytotoxicity assay kits (Merck; Product No. CELLPRO-RO, BioChain Institut, WST-1 Cell Proliferation Assay Kit). Abs sample = absorbance of the test sample at 440 nm

### 3.9. Protein Isolation and HSP70 Protein Content

Proteins were isolated using the method of Isaacson et al. [82], with minor modifications. The tissue (400 mg) was ground in a cold mortar in 4 mL of 10% TCA in acetone. The extracts were transferred to Eppendorf tubes and stored at −20 °C for 24 h. The extracts were then centrifuged for 30 min at 5000× *g*. The extracts were purified by adding 4 mL of cold acetone. The pellet washing was repeated twice, followed by centrifugation for 10 min at 4 °C, at a speed of 5000× *g*. The pellet was dried at room temperature and then suspended in a TBS buffer containing 250 mM Tris, 1.37 M NaCl. The HSP70 protein content was determined using ELISA kit (EIAab Science, Wuhan, China). Then, 100 μL of protein samples were applied to a 96-well plate and then incubated at 37 °C. Further steps were carried out following the manufacturer’s protocol, and the plate was incubated again at 37 °C for an hour. Next, the wells were washed again, the substrate was applied, and the reaction was carried out at 37 °C for 20 min. The absorbance at the 450 nm wavelength was measured. The sample Diluent solution was used as a blank.

### 3.10. Statistics

All the tests were carried out in triplicates. The results were analysed in the Statistica program using the ANOVA (univariate) test. The differences between the trials were analysed using Tukey’s post-hoc test at the significance level *p* ≤ 0.005.

## 4. Conclusions

Our studies firmly suggested that the tetracycline contamination of water leads to manifold disturbances in the metabolism of *Lemna minor* L., including: water balance; photosynthetic apparatus (chlorophyll); respiration (mitochondrial dehydrogenase activity); membrane lipid peroxidation; accumulation of free radicals and the activation of free radical scavenging mechanisms. On the other hand, duckweed shows a considerable capacity to recover from intoxication with modetate doses (up to 2.5 mM approx. 1 g × L^−1^) of tetracycline. A significant improvement in the physiological status of the plants was observed within one week of the transfer to a tetracycline-free medium. However, the damages to the mitochondria caused by high doses of tetracycline tended to accumulate, even after the plants were transferred to an antibiotic-free medium. A wide range of plant-stress responses were probed in the experiments described, so it was not possible to go deeply into the mechanism of each of them. However, the data obtained should be helpful for predicting the outcomes of transient, accidental contamination of water reservoirs with tetracycline, one of the most widespread antibiotic pollutants of water. The data should also provide a useful framework for similar analyses in other aquatic plants and for future, more in-depth analyses.

## Figures and Tables

**Table 1:** EC50 values for morphological in *Lemna minor* L. exposure (subjected to tetracycline) and recovery (resumed growth after 7 days of culture in antibiotic-free medium).

	EC50 Value (Exposure) [mM]	EC50 Value (Recovery) [mM]
Number of fronds	1.944	2.515
Fronds area	1.724	2.850
Fresh mass	1.431	2.447
Dry mass	3.825	6.715

**Table 2:** Chlorophyll a content in *Lemna minor* subjected to tetracycline treatment (exposure) and in the recovery (resumed growth after 7 days of culture in antibiotic free medium).

Tetracycline Concentration [mM] during 7 Days	Chlorophyll Concentration [M]	
	Exposure	Recovery
0	1.574 × 10^−5^	1.574 × 10^−5^
1	0.918 × 10^−5^	1.315 × 10^−5^
2.5	0.749 × 10^−5^	0.988 × 10^−5^
10	0.571 × 10^−5^	0.742 × 10^−5^

**Table 3:** EC50 values for catalase activity (CAT), ascorbate peroxidase (APX), pyrogallol peroxidase (PDX), glutathione reductase (GR) in *Lemna minor* subjected to tetracycline (exposure) and in the recovery (R) (resumed growth after 7 days of culture in antibiotic free-medium).

Antioxidant Enzymes	EC50 Value (Exposure) [mM]	EC50 Value (Recovery) [mM]
Catalase activity	2.711	6.506
Ascorbate peroxidase	2.173	5.231
Pyrogallol peroxidase	9.253	5.098
Glutathione reductase	1.612	3.002

## Data Availability

Not applicable.

## Supplementary Materials

The following are available online. Table S1: Antibiotic contents [μg × L^−1^] in river water, drinking water, groundwater, sea and lake water [83,84,85,86,87,88,89,90,91,92,93,94,95,96,97,98,99,100,101,102,103,104,105,106,107,108,109,110,111,112,113,114,115,116,117,118,119,120,121,122,123,124,125].

## Sample Availability

Not available.
