# Supplementary material for: Physiological and Biochemical Parameters of Common Duckweed Lemna minor after the Exposure to Tetracycline and the Recovery from This Stress
Source: Molecules. 2021 Nov 9;26(22):6765. doi: 10.3390/molecules26226765 (PMC8625026; doi:10.3390/molecules26226765)
Supplement: Supplementary file 1 [file molecules-26-06765-s001.zip › molecules-1409955-supplementary.pdf]

Physiological and Biochemical Parameters of Common Duckweed *Lemna minor* after the Exposure to Tetracycline and the Recovery from This Stress

Magdalena Krupka[1], Dariusz J. Michalczyk[1], Jūratė Žaltauskaitė[2], Gintarė Sujetovienė[2], Katarzyna Głowacka[1], Hanna Grajek[3], Marta Wierzbicka[1] and Agnieszka I. Piotrowicz-Cieślak[1]*

[1] Department of Plant Physiology, Genetics and Biotechnology, Faculty of Biology and Biotechnology, University of Warmia and Mazury in Olsztyn, Oczapowskiego 1A, 10-718 Olsztyn, Poland
[2] Department of Environmental Sciences, Vytautas Magnus University, Universiteto 10, 46265, Kaunas, Lithuania
[3] Department of Physics and Biophysics, Faculty of Food Science, University of Warmia and Mazury in Olsztyn, Oczapowskiego 4, 10-719 Olsztyn, Poland
*Correspondence: acieslak@uwm.edu.pl; (AP-C)

Table S1. Antibiotic contents [μg × L^{-1}] in river water, drinking water, groundwater, sea and lake water.

ANTIBIOTIC	CONCENTRATION	COUNTRY/REGION	REFERENCE
**RIVER WATER SAMPLES [μg × L^{-1}]**			
**Amoxicilin**	0.18 – 1.21	Brazil (Belem river)	[83]
**Azithromycin**	0.003 – 0.221	Portugal, Porto (Leca river)	[84]
	0.034 – 1.52	Poland (Vistula river)	[85]
	0.019	Spain (Ebro river)	[86]
	0.00767	China (Huangshui river)	[87]
	1.6	Spain (Mijares river)	[88]
	0.008 – 0.5	Brazil (Belem river)	[83]
**Chloramphenicol**	0.028	China (Huangpu river)	[89]
	0.00227	Brazil (Netravathi tropical river)	[90]
**Chlorotetracycline**	0.017	China (Huangpu river)	[89]
	0.059	Spain (Ebro river)	[86]
	n.d. – 0.1	China (Yangtze River)	[91]
	0.42	USA, river sample	[92]
**Ciprofloxacin**	0.115	Spain (Ebro river)	[86]
	0.004 – 0.277	Poland (Vistula river)	[85]
	n.d. – 0.339	Portugal, Porto (Leca river)	[84]
	1.1	Spain (Mijares river)	[88]
	n.d. – 0.329	Brazil (Diluvio river)	[93]
**Clindamycine**	0.0122 – 0.0127	Poland (Warta river)	[94]

	0.13	Spain (Mijares river)	[88]
**Enrofloxacin**	0.178	Spain (Ebro river)	[86]
**Erythromycin**	0.0157	Spain (Ebro river)	[86]
	0.0076 – 1.02	Poland (Vistula river)	[85]
	0.0166	China (Huangshui river)	[87]
	0.12	Spain (Mijares river)	[88]
	0.009-0.041	Vietnam (Mekong river)	[95]
**Levofloxacin**	0.0052 – 0.08	Poland (Vistula river)	[85]
**Norfloxacin**	0.94	Spain (Mijares river)	[88]
	0.029-0.292	Brazil (Diluvio river)	[93]
	0.163	France (Seine river)	[96]
**Ofloxacin**	n.d. – 0.12	Portugal, Porto (Leca river)	[84]
	0.11 – 0.68	Europe, France	[97]
**Oxytetracycline**	n.d – 0.1	China (Yangtze River)	[91]
	0.34	USA, river sample	[92]
**Penicillin G**	0.0121	China (Huangshui river)	[87]
	0.013-0.017	Poland (Warta river)	[94]
**Sulfamethoxazole**	0.014	China (Huangpu river)	[89]
	0.00117 – 0.552	China (Huangshui river)	[87]
	0.0059 – 1.77	Poland (Vistula river)	[85]
	0.029 - 0.0377	Poland (Warta river)	[94]
	0.00102	Brazil (Swarna tropical river)	[90]
	0.00056 – 0.00141	Brazil (Netravathi tropical river)	[90]
	0.2	Spain (Mijares river)	[88]
	1.09 – 1.32	Brazil (Belem river)	[83]
	0.0034 – 0.35	UK (Thames river)	[98]
	0.02 – 0.174	Vietnam (Mekong river)	[95]
	0.544	France (Seine river)	[96]
	n.d. – 13.8	Kenya (Nairobi river)	[99]
**Tetracycline**	n.d. – 0.0315	China (Hong Kong rivers)	[100]
	n.d. – 0.0714	China (Yangtze River)	[91]
	0.11	USA	[92]
**LAKE WATER SAMPLES [μg × L^{-1}]**			
**Amoxicilin**	n.d. – 1.12	Vietnam, Hanoi	[101]
**Azithromycin**	0.004 – 0.089	Vietnam, Hanoi	[101]
	0.00314 – 0.9	China, Wuhan	[102]
**Ciprofloxacin**	0.017 – 0.112	China (Bosteng lake)	[103]
	n.d. – 0.169	Vietnam, Hanoi	[101]
	n.d. – 0.043	China (Taihu lake)	[104]
	n.d. – 0.0086	China (Poyang lake)	[105]
**Enrofloxacin**	n.d. – 0.015	China (Bosteng lake)	[103]

	n.d. – 0.0055	China (Poyang lake)	[105]
	0.005 – 0.169	Vietnam, Hanoi	[101]
**Erythromycin**	n.d. – 0.624	China (Taihu lake)	[104]
	0.107	China (Poyang lake)	[105]
	n.d. – 0.741	Vietnam, Hanoi	[101]
**Norfloxacin**	0.00049 – 0.045	China, Wuhan	[102]
**Ofloxacin**	n.d. – 0.0828	China (Taihu lake)	[104]
	0.021	USA (Michigan lake)	[106]
	0.021	China, Wuhan	[102]
**Penicillin G**	0.00288 – 0.037	China (Bosteng lake)	[103]
**Sulfamethoxazole**	n.d. – 0.114	China (Taihu lake)	[104]
	n.d. – 0.0014	China (Poyang lake)	[105]
	n.d. – 0.077	USA (Michigan lake)	[106]
	0.00028 – 1.2	USA (Lead lake)	[107]
	0.108 – 3.5	Vietnam, Hanoi	[101]
	0.0012 – 0.016	China, Wuhan	[102]
**Trimetophrim**	n.d. – 0.12	USA (Lead lake)	[107]
	n.d. – 0.0633	Canada (Ontario lake)	[108]
**SEA WATER SAMPLES [μg × L^{-1}]**			
**Amoxicilin**	n.d. – 0.076	China (South China Sea)	[109]
**Azithromycin**	n.d. – 0.138	China (Yellow sea)	[110]
**Chloramphenicol**	n.d. – 0.073	China (Yellow sea)	[110]
	0.2 – 15.6	Tunisia (Mediterranean Sea)	[111]
**Enrofloxacin**	n.d. – 0.121	China (Yellow sea)	[110]
**Norfloxacin**	n.d. – 0.021	China (Yellow sea)	[110]
	n.d. – 0.027	China (South China Sea)	[109]
**Ofloxacin**	n.d. – 0.497	China (Yellow sea)	[110]
	0.008 – 0.6	China (South China Sea)	[109]
**Penicillin G**	n.d. – 0.00118	China (Yellow sea)	[110]
**Sulfamethoxazole**	n.d. – 0.0481	China (Yellow sea)	[110]
	n.d. – 0.0475	China (South China Sea)	[109]
**Trimetophrim**	0.0014 – 0.095	China (Yellow sea)	[110]
**GROUNDWATER SAMPLES [μg × L^{-1}]**			
**Chloramphenicol**	0.026	China	[112]
**Ciprofloxacin**	0.1	China	[112]
	0.7	India	[113]
**Erythromycin**	0.345	China	[112]
**Lincomycin**	0.320	USA	[114]
	0.86	China	[112]
**Norfloxacin**	0.4	China	[112]
**Ofloxacin**	1.19	China	[112]
**Sulfamethoxazole**	1.11	USA	[114]
	0.312	Spain, Catalonia	[115]
	0.458	USA, California	[116]

	≤0.1	Germany	[117]
	0.018	Netherlands	[20]
**DRINKING WATER SAMPLES [μg × L^{-1}]**			
**Azithromycin**	0.193	Poland (tap water sample)	[85]
	0.00008 – 0.00055	Germany (drinking water reservoir system)	[118]
	0.00212 – 0.00902	China, Nanjing	[119]
**Ciprofloxacin**	0.27	China (tap water sample)	[120]
**Clindamycin**	0.00004 – 0.00034	Germany (drinking water reservoir system)	[118]
**Enrofloxacin**	0.00038 – 0.00144	China, Nanjing	[119]
**Erythromycin**	0.057	Poland (tap water sample)	[85]
**Florfenicol**	0.011	China (tap water sample)	[121]
	0.0011 – 0.0077	China (large-scale drinking water source, Yangtze River)	[122]
**Norfloxacin**	0.00096	China, Nanjing	[119]
**Oxytetracycline**	0.0006	China, Hong-Kong (tap water sample)	[123]
**Sulfadiazine**	0.00008 – 0.00016	China, Nanjing	[119]
**Sulfamethoxazole**	0.000164	Spain (bottled water sample)	[124]
	0.00039	USA	[125]
	0.00003 – 0.0004	Germany (drinking water reservoir system)	[118]
	0.000198	China, Nanjing	[119]
	0.00428	China (large-scale drinking water source, Yangtze River)	[122]
**Tetracycline**	n.d. – 0.027	China (drinking water source, Yangtze River)	[21]

n.d. – not detected
